# Generative adversarial network enables rapid and robust fluorescence lifetime image analysis in live cells

**DOI:** 10.1038/s42003-021-02938-w

**Published:** 2022-01-11

**Authors:** Yuan-I Chen, Yin-Jui Chang, Shih-Chu Liao, Trung Duc Nguyen, Jianchen Yang, Yu-An Kuo, Soonwoo Hong, Yen-Liang Liu, H. Grady Rylander, Samantha R. Santacruz, Thomas E. Yankeelov, Hsin-Chih Yeh

**Affiliations:** 1 Department of Biomedical Engineering, The University of Texas at Austin, Austin, TX 78712 USA; 2 ISS, Inc., 1602 Newton Drive, Champaign, IL 61822 USA; 3 Master Program for Biomedical Engineering, China Medical University, Taichung, 406040 Taiwan; 4 Research Center for Cancer Biology, China Medical University, Taichung, 406040 Taiwan; 5 Institute for Neuroscience, The University of Texas at Austin, Austin, TX 78712 USA; 6 Department of Electrical and Computer Engineering, The University of Texas at Austin, Austin, TX 78712 USA; 7 Oden Institute for Computational Engineering and Sciences, The University of Texas at Austin, Austin, TX 78712 USA; 8 Department of Diagnostic Medicine, The University of Texas at Austin, Austin, TX 78712 USA; 9 Department of Oncology, The University of Texas at Austin, Austin, TX 78712 USA; 10 Livestrong Cancer Institutes, The University of Texas at Austin, Austin, TX 78712 USA; 11 Department of Imaging Physics, The University of Texas MD Anderson Cancer Center, Houston, TX 77030 USA; 12 Texas Materials Institute, The University of Texas at Austin, Austin, TX 78712 USA

**Keywords:** Fluorescence imaging, Software

## Abstract

Fluorescence lifetime imaging microscopy (FLIM) is a powerful tool to quantify molecular compositions and study molecular states in complex cellular environment as the lifetime readings are not biased by fluorophore concentration or excitation power. However, the current methods to generate FLIM images are either computationally intensive or unreliable when the number of photons acquired at each pixel is low. Here we introduce a new deep learning-based method termed *flimGANE* (fluorescence lifetime imaging based on Generative Adversarial Network Estimation) that can rapidly generate accurate and high-quality FLIM images even in the photon-starved conditions. We demonstrated our model is up to 2,800 times faster than the gold standard time-domain maximum likelihood estimation (*TD_MLE*) and that *flimGANE* provides a more accurate analysis of low-photon-count histograms in barcode identification, cellular structure visualization, Förster resonance energy transfer characterization, and metabolic state analysis in live cells. With its advantages in speed and reliability, *flimGANE* is particularly useful in fundamental biological research and clinical applications, where high-speed analysis is critical.

## Abstract (web summary)

In this study, Chen et al. introduced a new deep learning-based method termed *flimGANE* to rapidly generate accurate and high-quality FLIM images even in the photon-starved conditions. *flimGANE* is particularly useful in fundamental biological research and clinical applications.

## Introduction

Using fluorescence decay rate as the contrast mechanism, fluorescence lifetime imaging microscopy (FLIM) is a powerful quantitative tool for studying cell and tissue biology^1–3^, allowing us to monitor the pH^4^, viscosity^5^, temperature^6^, oxygen content^7^, metabolic state^8^ and functional property of a biomarker^9^ inside live cells or tissues. Depending on the intrinsic property of fluorophore, FLIM images are not skewed by fluorophore concentration and excitation power, eliminating the biases introduced by the traditional intensity-based images^2^. Combined with the Förster resonance energy transfer (FRET) sensors^10^, FLIM can probe Ca^2+^ concentration^11^, glucose concentration^12^, and protein-protein interactions^13^, without the need to measure acceptor’s fluorescence^14^. Whereas FLIM offers many unique advantages in quantifying molecular interactions^15^ and chemical environments^16^ in biological or chemical samples, fluorescence lifetime analysis is a slow process with results often impaired by fitting errors. Adopted from disparate disciplines, various fluorescence lifetime estimation methods, such as curve fitting (least-squares fitting^17^, maximum likelihood estimation^18^, global analysis^19^, and Bayesian analysis^20^), phasor analysis^21,22^, and deconvolution analysis (stretched exponential analysis^23^ and Laguerre deconvolution^24^) have been developed to infer the lifetime of interest. However, these methods are often limited by long computation times, poor accuracy particularly in the low-light conditions, and invalid initial assumptions of decay parameters. Although deep learning methods, such as artificial neural network (ANN)^25^ or convolutional neural network (CNN)^26,27^, have been employed to achieve rapid fluorescence lifetime analysis in the medium-photon-count conditions (200–500 photon counts per pixel), other deep learning algorithms may further improve the reliability in analyzing the low-photon-count (100–200 photon counts per pixel) or even ultralow-photon-count data (50–100 photon counts per pixel) for live-cell imaging.

Here we demonstrate a new fluorescence lifetime imaging method based on Generative Adversarial Network Estimation (*flimGANE*) that can provide fast, fit-free, accurate, and high-quality FLIM images even under the photon-starved conditions (50–200 photon counts per pixel). GAN is one of the frameworks for evaluating generative models *via* an adversarial process^28^, which has been adopted to improve astronomical images^29,30^, transform images across different modalities^29,31^, and design drugs that target specific signaling molecules^32^. While GAN-based algorithms have recently drawn much attention for inferring photo-realistic natural images^33^, they have not been used to generate high-quality FLIM images based on the fluorescence decays collected by a laser scanning confocal microscope. Our *flimGANE* method is adapted from the Wasserstein GAN algorithm^34^ (WGAN), where the generator (*G*) is trained to produce an “artificial” high-photon-count fluorescence decay histogram based on a low-photon-count input, while the discriminator (*D)* distinguishes the artificial decay histogram from the ground truth (which can be a simulated dataset or a decay histogram collected under strong excitation). As a minimax two-player game, the training procedure for *G* is to maximize the probability of *D* making a mistake^28^, eventually leading to the production of very realistic, artificial high-photon-count decay histograms that can be used to generate a high-quality FLIM image. Using a well-trained generator (*G*) and an estimator (*E*), we can reliably map a low-quality decay histogram to a high-quality counterpart, and eventually to the three lifetime parameters (*α*_*1*_, *τ*_*1*_, and *τ*_*2*_) within 0.32 ms/pixel using a CPU. Without the need of curve fitting based on initial guesses, our *flimGANE* method is up to 258-fold faster than the time-domain least-squares estimation (*TD_LSE*^35,36^) and 2,800-fold faster than the time-domain maximum likelihood estimation (*TD_MLE*^37,38^) in generating a 512 × 512 FLIM image. While almost all commercial FLIM analysis tools are based on *TD_LSE*, using the least-squares estimator to analyze Poisson-distributed data is known to lead to biases^39^, making *TD_MLE* the gold standard for FLIM analysis by many researchers^18^. Our *flimGANE* can provide similar FLIM image quality as *TD_MLE*, but much faster. Recently, field-programmable gate array (*FPGA*)-based *MLE* has been demonstrated for lifetime analysis, reaching an ultrahigh analysis speed^40^. However, *FPGA-MLE* needs much more effort in hardware development and programing to be implemented in an existing optical system.

Overcoming a number of hardware limitations in the classical analog frequency-domain approach, the digital frequency-domain (*DFD*) lifetime measurement method has substantially increased the FLIM acquisition speed^21,22,41^. The acquired *DFD* data at each pixel, termed a cross-correlation phase histogram, can lead to a phasor plot with multiple harmonic frequencies^41^. From such a phasor plot, modulation ratio and phase angle at each harmonic frequency can be obtained, which are then fitted with a least-squares estimator (*LSE*) to generate a lifetime at each pixel (termed the *DFD_LSE* method). Our *flimGANE* not only runs nearly 12-fold faster than *DFD_LSE* but also produces more accurate quantitative results in all photon-count conditions. Sharper structural images of *Convallaria* and live HeLa cells are also achieved by *flimGANE*. Whereas the lowest number of photons needed for reliable estimation of a fluorescence lifetime by *TD_MLE* is about 100 photons^42^, *flimGANE* performs consistently well with a photon count as low as 50 per pixel in our simulations. Moreover, *flimGANE* improves the energy transfer efficiency estimate of a glucose FRET sensor, leading to a more accurate glucose concentration measurement in live HeLa cells. Providing both efficiency and reliability in analyzing low-photon-count decays, our *flimGANE* method represents an important step forward towards real-time FLIM.

## Results

### Training the generative adversarial network in *flimGANE*

**Fig. 1:**
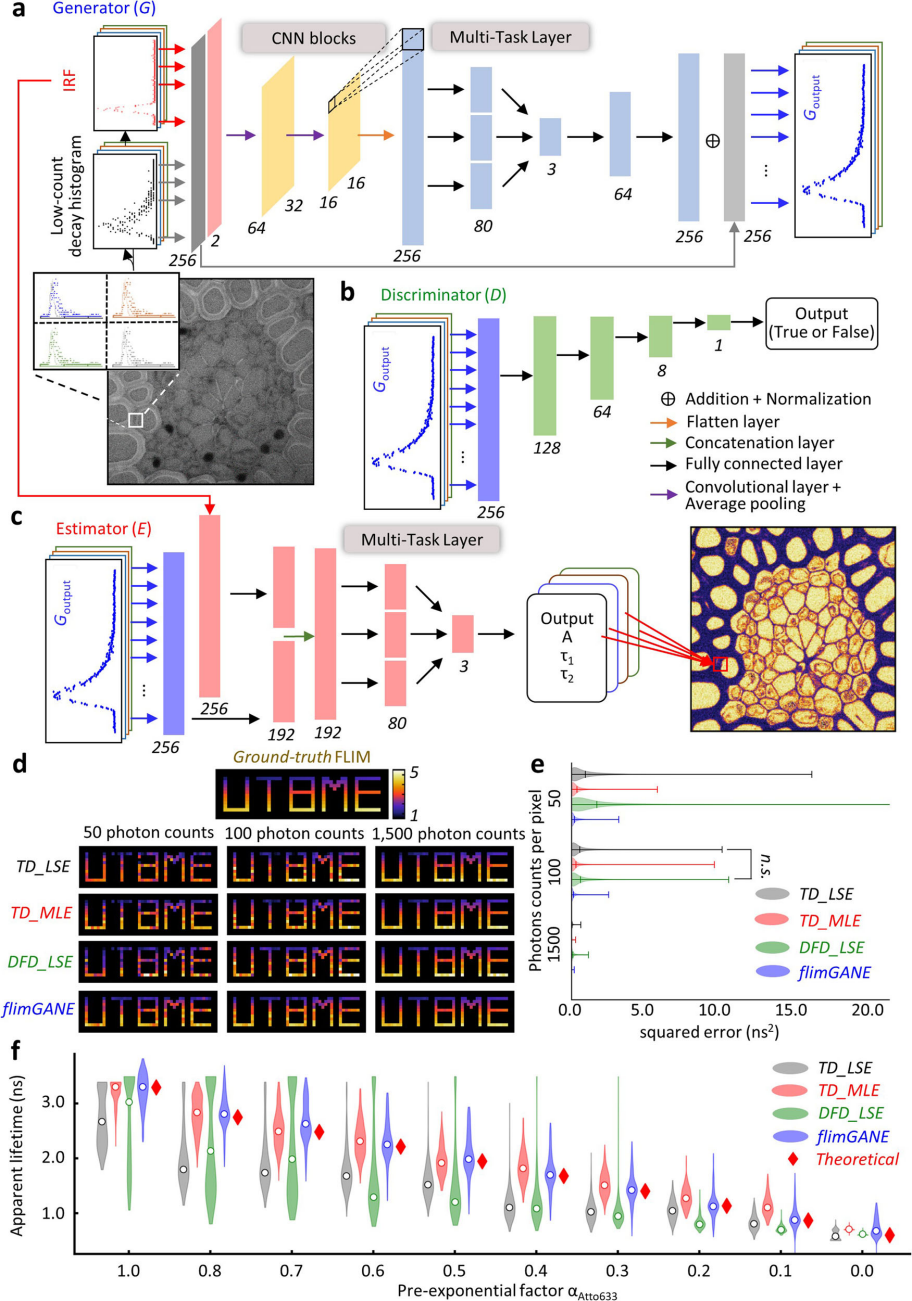
*flimGANE* (fluorescence lifetime imaging based on Generative Adversarial Network Estimation) is a rapid and accurate method to generate fluorescence lifetime microscopy (FLIM) images. **a**–**c** Schematic of the deep learning framework for *flimGANE* architecture. **a** Generator (*G*) is used to transform the acquired decay curve into an artificial high-photon-count decay. It comprises two CNN blocks, each of which is made up of one convolutional layer followed by an average pooling layer of stride four. The CNN section is followed by a flatten layer. Then a multi-task layer converts data into virtual lifetime parameters, followed by two fully connected layers. Skip connection is used to pass data between layers of the same level. **b** Discriminator (*D*) consists of four fully connected layers. **c** Estimator (*E*) comprises a partially and a fully connected layers followed by a multi-task layer to map the high-photon-count decay curve into lifetime parameters. **d** Comparison of FLIM images generated by different methods (*n* = 1340 pixels). **e** Comparison of squared errors by different methods. Under the ultra-low-photon-count condition (50 counts per pixel), *flimGANE* provides the best estimation. Under the low-photon-count condition (100 counts), *TD_LSE* and *DFD_LSE* fail to generate accurate FLIM images. Under the high-photon-count condition (1500 counts), all FLIM images match well with the ground truth. *TD_LSE* result exhibits no significant difference from *DFD_LSE* result under the low-photon-count condition based on the two-tailed *t* test. **f** *flimGANE* successfully characterizes the apparent lifetimes of the two-dye mixtures (stock solution: 3 μM Cy5-NHS ester and 7 μM Atto633 in DI water). The mean values obtained from Gaussian fitting are indicated as white solid circles.

Based on the Wasserstein GAN framework (see Supplementary Methods), *flimGANE* is designed to analyze one- or two-component fluorescence decays under the photon-starved conditions (Fig. 1). There are two ways to generate a dataset of ground-truth lifetime histograms for training *G* and *D—*either by creating a decay dataset using Monte Carlo (MC) simulations or by acquiring an experimental dataset from standard organic fluorophores under high excitation power. The inputs of *G* are degraded data from the ground truths, which can be obtained by running simulations at a low-emission rate or by recollecting experimental data under low excitation power.

We started our network training using an MC simulation dataset (Supplementary Fig. 1). A Python program was employed to simulate the photon collection process in the counting device with 256-time bins, following the probability mass function (pmf) numerically calculated by the convolution of an experimentally obtained instrument response function (IRF) and a theoretical two-component decay model (*α*_*1*_, *τ*_*1*_, 1–*α*_*1*_, and *τ*_*2*_) at a selected emission rate (*rate*)^43^. Depending on the fluorophores that users want to image, proper *α*_*1*_, *τ*_*1*_, *τ*_*2*_ and *rate* parameters that span the range of interest could be selected (Supplementary Tables 1 and 2), generating about 600 normalized ground truths and 300,000 degraded decays for training *G* and *D*. The adversarial network training was completed in 6.1 h (Fig. 1a, b; Supplementary Fig. 2). The normalized degraded decay was transformed into the normalized “ground-truth mimicking” histogram, termed *G*_*output*_, with one-to-one correspondence (Supplementary Fig. 3), within 0.17 ms. Such a *G*_*output*_ was indistinguishable by *D* from the ground truth dataset. *E*, which was separately trained on the ground truths and completed in 0.1 h, was then employed to extract the key lifetime parameters (*α*_*1*_, *τ*_*1*_, and *τ*_*2*_) from the *G*_*output*_ within 0.15 ms (Fig. 1c; Supplementary Fig. 2). In this process, *flimGANE* did not take information from the surrounding pixels into the current pixel.

**Fig. 2:**
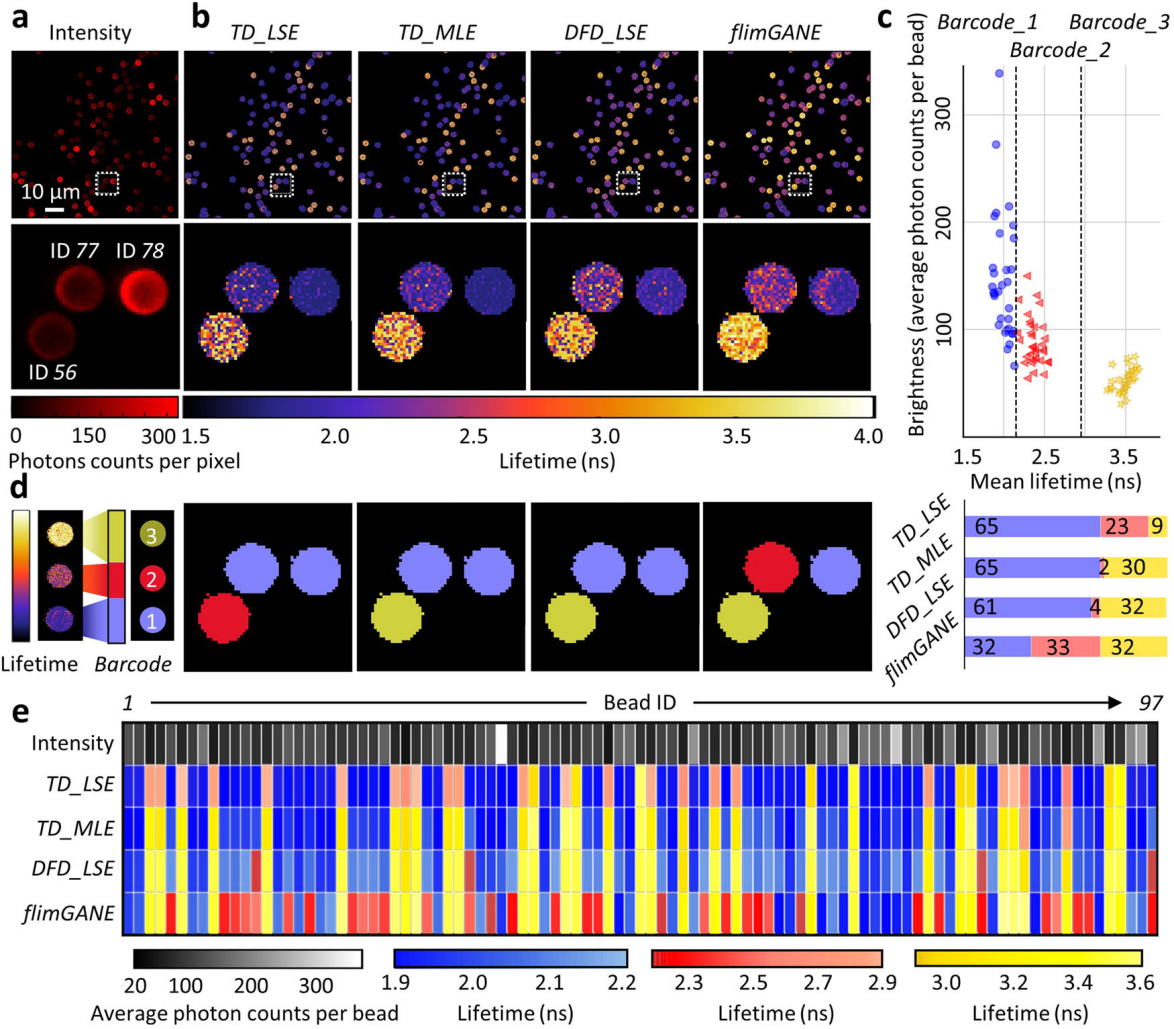
*flimGANE* accurately classifies the three fluorescence lifetime barcode beads. **a** Intensity contrast images show 3-µm fluorescent beads with various brightness. **b** FLIM images obtained from *TD_LSE*, *TD_MLE*, *DFD_LSE*, and *flimGANE* discriminate fluorescence lifetime barcode beads. **c** Mean lifetimes versus brightness plot of 97 beads shows the intensity differences of the three barcodes and the cutoff lifetimes (2.15 and 2.95 ns) for barcode identification. **d** Barcode classification results by the four methods indicate that only *flimGANE* can correctly identify the three barcodes and restore the correct barcode ratio (1:1:1). **e** Classification results of all 97 beads show that many *barcode_2* beads are misidentified as *barcode_1* beads by *TD_LSE*, *TD_MLE,* and *DFD_LSE*, while the identification of *barcode_3* beads is more reliable among these methods (except for *TD_LSE*, which performs poorly under the low-light condition).

**Fig. 3:**
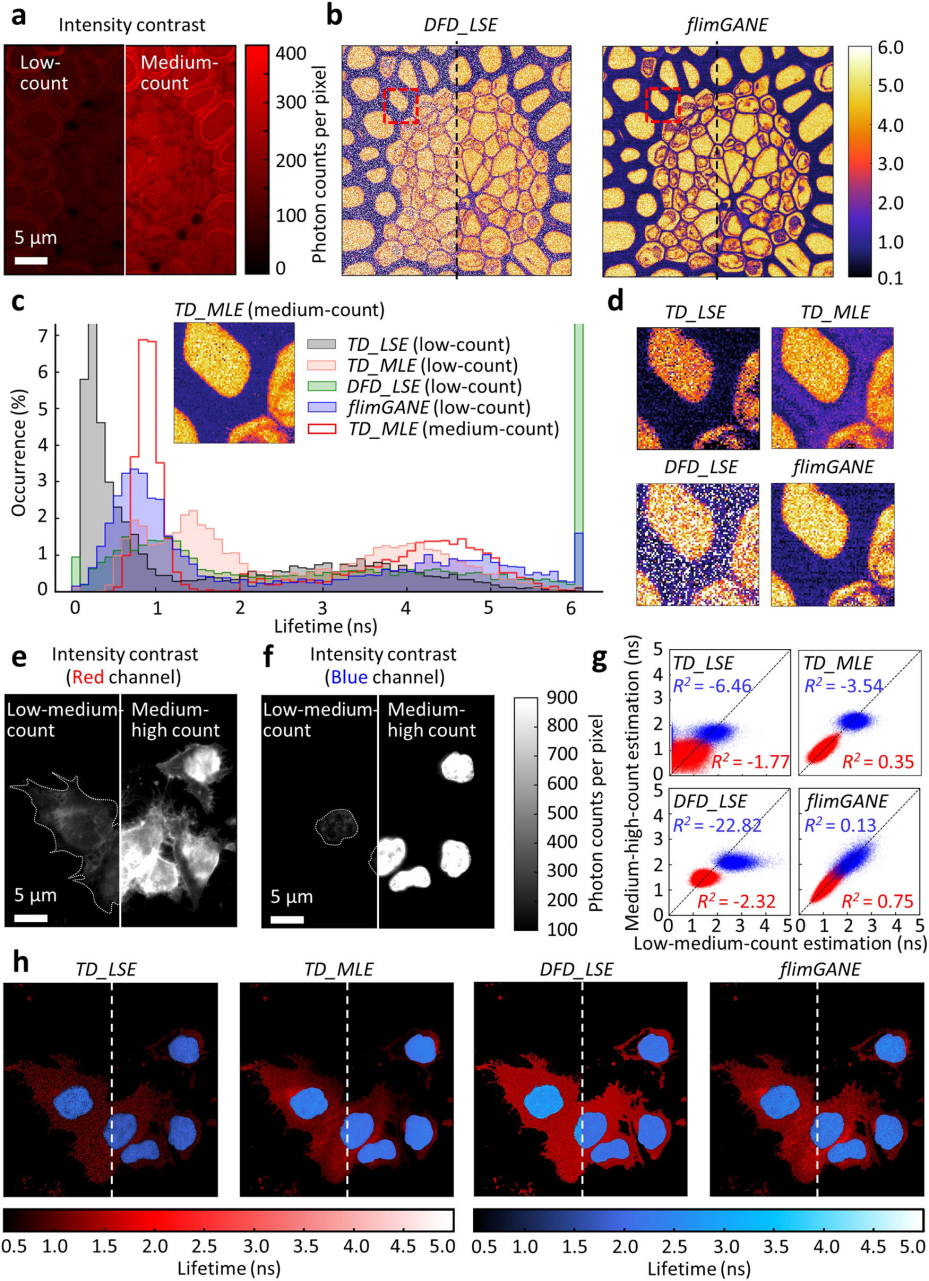
*flimGANE* provides sharper structural images of *Convallaria* and HeLa cells. **a** Images of *Convallaria* acquired at two different intensity levels (left: 50–150 counts per pixel and right: 200–400 counts). **b** FLIM image generated by *flimGANE* is sharper and provides more structural details than image from *DFD_LSE*. **c** Lifetime histograms obtained from *TD_LSE*, *TD_MLE*, *DFD_LSE*, and *flimGANE *show that *flimGANE* histogram most resembles that of the standard (*TD_MLE* at the medium-photon-count condition). **d** A zoom-in of red box in b. *flimGANE* clearly reveals more structural details than other methods. **e** Intensity contrast images of plasma membrane of live HeLa cells in red channel (685/40 nm) under two photon-count conditions. Dash lines represent the contours of live cells. **f** Intensity contrast images of nuclei of live HeLa cells in blue channel (494/34 nm) under two photon-count conditions. **g** 2D scatter plots of lifetime acquired at two photon-count conditions. *flimGANE* provided more consistent estimates under both conditions. The coefficients of determination, *R*^*2*^, ranging from −∞ to 1.00, are utilized to evaluate the consistency. **h** Overlay of FLIM images in red and blue channels (left: low-medium-photon-count; right: medium-high-photon-count).

**Fig. 4:**
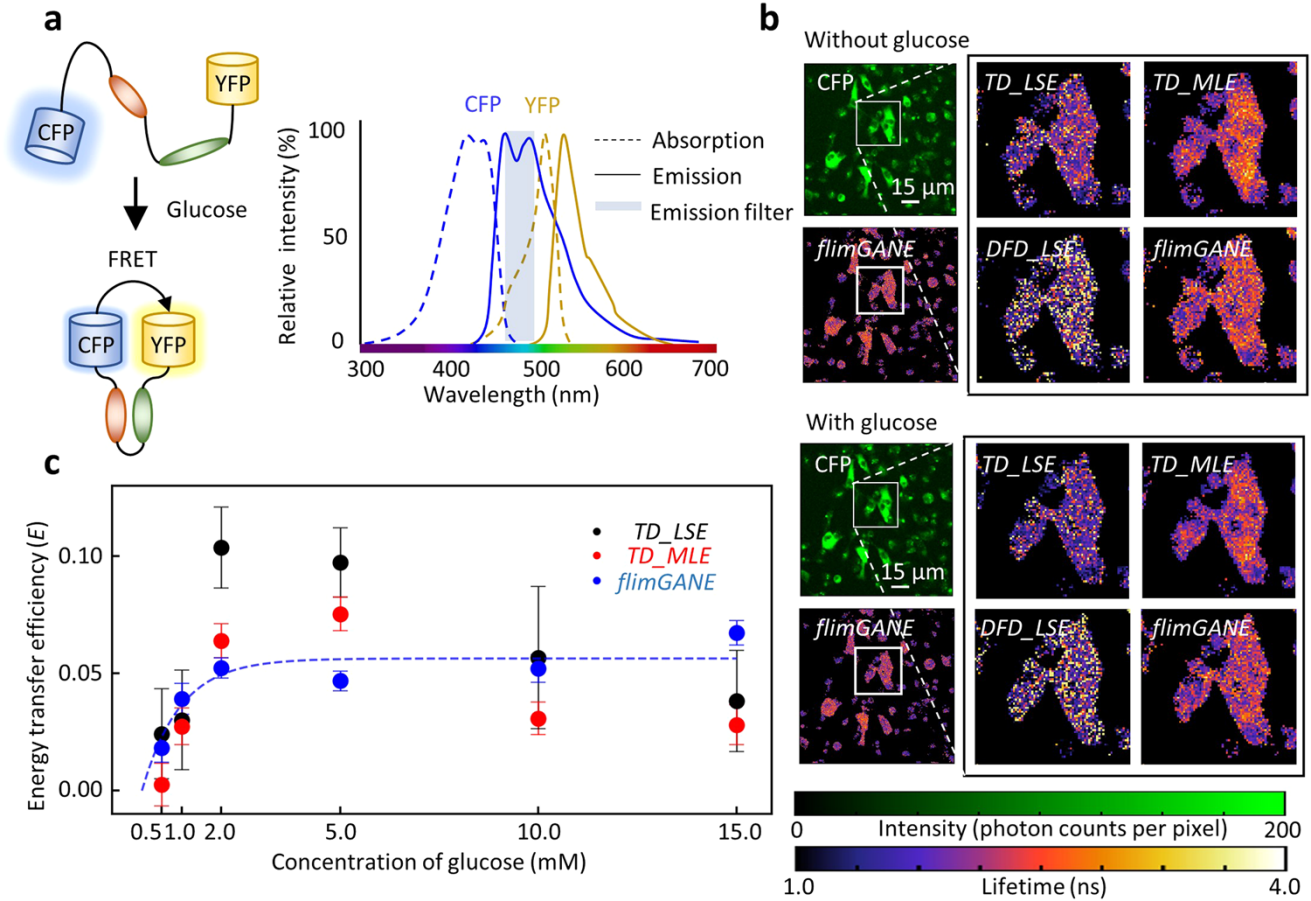
*flimGANE* enables accurate  FRET characterization from low-photon-count data. **a** Schematic of CFP-g-YFP FRET pair interacting with glucose and normalized excitation and emission spectra of CFP and YFP. **b** Intensity contrast and FLIM images of CFP generated by *TD_LSE*, *TD_MLE*, *DFD_LSE* and *flimGANE* before and after adding 2 mM glucose. **c** Energy transfer efficiency, *E*, versus  glucose concentration (error bars, standard deviation errors on the parameter estimate, *n* = 1507~6824 pixels). The *flimGANE* FRET data can be well fitted by a sigmoidal curve (R2 = 0.92).

**Fig. 5:**
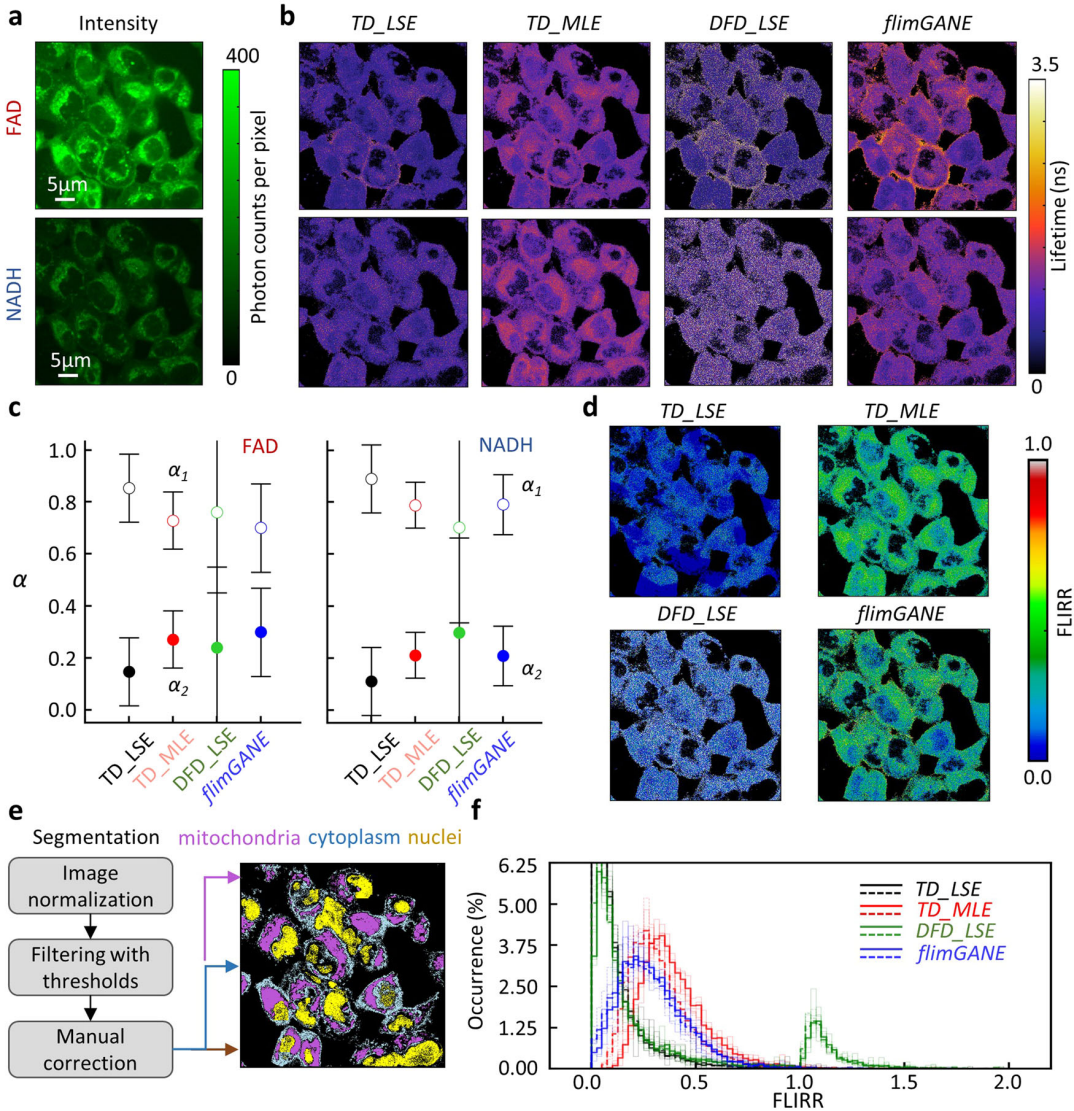
*flimGANE* enables precise metabolism quantification from low-photon-count autofluorescence data in live HeLa cells. **a** Intensity contrast images of FAD and NAD(P)H. **b** FLIM images of FAD and NAD(P)H generated by *TD_LSE*, *TD_MLE*, *DFD_LSE,* and *flimGANE.* **c** Pre-exponential factors (α_1_ and α_2_ = 1-α_1_, where α_1_ is the fraction of short lifetime component) of FAD and NAD(P)H obtained by different methods (error bars, standard deviation, n = 160,000 pixels). **d** FLIRR images show that *flimGANE* result best matches with *TD_MLE* result. **e** Intensity contrast images from **a** are normalized and segmented for mitochondria, cytoplasm, and nuclei. **f** Comparison of FLIRR results obtained from *TD_LSE*, *TD_MLE*, *DFD_LSE*, and *flimGANE* (solid line: based on the mitochondria images; dashed line: based on the whole-cell images without nuclei; *n* = 5 cells).

To demonstrate the reliability of our *flimGANE* method, we created ten sets of 14 × 47 “*UTBME*” FLIM images *in silico* (independently generated, not used in the training process) at three-photon emission rates (50, 100, and 1500 photons per pixel). At 1500 photons per pixel, all four methods (*TD_LSE*, *TD_MLE*, *DFD_LSE* and *flimGANE*) generated high-fidelity FLIM images (based on the apparent lifetime, *τ*_*α*_ = *α*_*1*_*τ*_*1*_ + (1 − *α*_*1*_)*τ*_*2*_), with mean-squared errors (MSE) less than 0.10 ns^2^ (*n* = 1,340 pixels). At 150 photons per pixel, *flimGANE* had similar performance as *TD_MLE* (MSE were both less than 0.20 ns^2^); however, *flimGANE* clearly outperformed *TD_LSE*, *TD_MLE*, and *DFD_LSE* at 50 photons per pixel (0.21 vs. 0.91, 0.36 and 1.65 ns^2^, respectively; Fig. 1d, e, Supplementary Figs. 4–6, and Supplementary Tables 3–6). CPU-based speed analysis showed that *flimGANE* was up to 258 and 2,800 times faster than *TD_LSE* and *TD_MLE*, respectively (*flimGANE—*0.32 ms per pixel, *TD_LSE—*82.40 ms, *TD_MLE—*906.37 ms; Supplementary Table 7). While *DFD_LSE* offered a decent speed in generating FLIM images (3.94 ms per pixel), its accuracy was worse than that of *flimGANE* (Figs. 2–5**)**. In contrast, being a computationally intensive method, *TD_MLE* offered the accuracy, but not the speed. Only *flimGANE* could provide both speed and accuracy in generating FLIM images. In addition, the MLE method became unreliable in the ultra-low-photon-count condition (50–100 photons per pixel), while *flimGANE* still provided a reasonable result.

To obtain accurate FLIM images, the IRF of the imaging system, which is mainly decided by the width of the laser pulse and the timing dispersion of detector, should be carefully considered during lifetime estimation. While the full width at half maximum (FWHM) of IRF is stable in most of the commercial FLIM imaging systems (detector time jittering within 35–500 ps^44^), users often observe that the delay between the single-photon detector output and the photon-counting electronics input varies from day to day, possibly due to the instability of the photon-counting electronics caused by radio-frequency interference, laser lock instability, and temperature fluctuation. Such delay changes cause the onsets of the decays to drift, deteriorating the *flimGANE* analysis results. A preprocessing step, termed Center of Mass Evaluation (CoME), is thus introduced to adjust (or standardize) the temporal location of the onset of experimental decays (Supplementary Figs. 7–9). After preprocessing, the apparent lifetimes estimated by *flimGANE* are found free of onset-delay bias.

To prove the reliability of *flimGANE* in estimating an apparent fluorescence lifetime from a mixture, two fluorophores, Cy5-NHS ester (*τ*_*1*_ = 0.60 ns) and Atto633 (*τ*_*2*_ = 3.30 ns), were mixed at different ratios, creating ten samples of distinct apparent fluorescence lifetimes (*τ*_*α*_) ranging from 0.60 to 3.30 ns. Here *τ*_*1*_ and *τ*_*2*_ were measured from the pure dye solutions and estimated by *TD_MLE*, whereas the theoretical apparent lifetime *τ*_*αT*_ was predicted by the equation *τ*_*αT*_ = *τ*_*1*_*α*_*1*_ + *τ*_*2*_(1 − *α*_*1*_). *α*_*1*_, the pre-exponential factor^45^, was derived from the relative brightness of the two dyes and their molar ratio^41^ (see Supplementary Methods). When analyzing 256 × 256-pixel images with emission rates fluctuating between 80 and 200 photons per pixel, *flimGANE* and *TD_MLE* produced the most accurate *τ*_*α*_ estimates among the four methods (Fig. 1f, and Supplementary Table 8). *TD_LSE* and *DFD_LSE* performed poorly in this low-light, two-dye mixture experiment.

### Discriminating fluorescence lifetime barcode beads

We then tested *flimGANE* in discriminating the fluorescence lifetime barcodes. To create fluorescence lifetime barcodes, biotinylated Cy5- and Atto633-labeled DNA probes were mixed at three different ratios, Cy5-DNA:Atto633-DNA = 1:0 (*barcode_1*, expecting lifetime 1.90 ns); 1:1 (*barcode_2*, 2.40 ns) and 0:1 (*barcode_3*, 3.50 ns), and separately conjugated to streptavidin-coated polystyrene beads (3–4 μm in size). It was noted that the lifetime of Cy5-DNA (1.90 ns) is different from that of Cy5-NHS ester (0.60 ns). Similarly, the lifetime of Atto633-DNA (3.50 ns) is different from that of Atto633 (3.30 ns). A coverslip coated with the three barcode beads (at equal molar concentration) was scanned by the ISS Alba v5 confocal microscopic system (equipped with a 20 MHz 635 nm diode laser for excitation and a *FastFLIM* module for *DFD* acquisition^41^) for 31 s, generating 512 × 512-pixel *DFD* data with photon counts ranging from 50-300 per pixel on the beads (Fig. 2a). The acquired *DFD* data (i.e., cross-correlation phase histograms^41^) were converted into time decays for *flimGANE*, *TD_LSE,* and *TD_MLE* analysis (Fig. 2b). Each barcode bead was registered by ImageJ ROI manager and assigned an ID number (Supplementary Fig. 10). An apparent lifetime was assigned to each pixel on the bead (~292 pixels) and lifetimes of all pixels were plotted in a histogram. The mean lifetime for the bead was determined by the Gaussian fitting of the histogram. After examining 97 beads, we chose the midpoint lifetimes (2.15 and 2.95 ns) to be the cutoffs for barcode identification (Fig. 2c) and assigned pseudocolors to the beads (Fig. 2d). It was clear to see that *flimGANE* is the only method that can correctly identify the three barcodes and restore the 1:1:1 barcode ratio, while other methods often misidentified the barcodes (Fig. 2e). Whereas it was a general trend that beads with more Atto633-DNA are dimmer, possibly due to stronger self-quenching, brightness alone could not classify the three barcodes (Fig. 2c, Supplementary Fig. 11, Supplementary Table 9). It was noted that the brightness of *barcode_1* beads could vary by sixfold, but the coefficient of variance (CV) of the *barcode_1* lifetimes was only 0.06, making lifetime a better metric to differentiate barcodes. Barcode discrimination by other MLE-based approaches were provided in Supplementary Fig. 12. Whereas the analysis speed of *MLE-pattern matching*^46^ was much faster than that of *flimGANE* (0.08 vs. 0.32 ms/pixel), *flimGANE* still had the best barcode discrimination accuracy.

### Visualizing cellular structures of *Convallaria* and HeLa cells

The *DFD* fluorescence data of *Convallaria* (lily of the valley) and live HeLa cells, acquired under the low- and medium-photon-count conditions (Fig. 3a), were analyzed by *DFD_LSE* and *flimGANE* (Fig. 3b), where *TD_MLE* (in the medium-photon-count condition, ~243 photons per pixel) served as the standard for comparison. The histogram clearly showed two characteristic lifetimes (0.90 ± 0.13 ns; 4.84 ± 1.20 ns) in the *Convallaria* sample (Fig. 3c). As *TD_MLE* with medium-photon counts had all lifetime estimates within 0.0–6.0 ns range, we limited the upper bound of the lifetime estimates to be 6.0 ns. Those 6.0 ns pixels were given the white pseudocolor and regarded as failed pixels in the FLIM images (Fig. 3d). A large number of failed pixels were seen in the *DFD_LSE* images (37% and 25% for the low- and medium-count images, respectively; Fig. 3b), deteriorating the visualization of structure details in the *Convallaria* sample. In contrast, there were very few failed pixels in the *flimGANE* images under the ultra-low-light condition (~83 photons per pixel), making them most resemble the *TD_MLE* images under the medium-light condition and provide better visualization of the structure details (Fig. 3c, d). The structure similarity index (SSIM)^47^ indicated that the *flimGANE* images were 73% more similar to the gold standard *TD_MLE* images than those generated by *DFD_LSE* (*flimGANE* −0.88, *DFD_LSE* −0.51; Supplementary Table 10), and visual information fidelity (VIF)^48^ showed that the *flimGANE* images were 1.44-fold higher than those reconstructed by *DFD_LSE* (*flimGANE* – 0.22, *DFD_LSE* – 0.09; Supplementary Table 10).

In the live HeLa cell sample, nuclei and membranes were stained with Hoechst and CellMask^TM^ Red and excited by 405 nm and 635 nm diode lasers, respectively. The contours of nuclei and cell membranes could not be clearly defined by the intensity-based images even under the low-light condition (Fig. 3e, f). Although FLIM overlay images allowed us to better visualize structural details in HeLa cells, the lifetime estimates could be biased when there were ~180 photons per pixel (low-light condition; *TD_LSE* and *DFD_LSE* images in Fig. 3h). Using the medium-high-count *TD_MLE* images (~600 photons per pixel) as the standard for comparison, *flimGANE* clearly outperformed *TD_LSE* and *DFD_LSE* in producing images that resemble the standard under the medium-light condition (Fig. 3h; Supplementary Table 11). Interestingly, when scrutinizing the assigned lifetime at each pixel, we found not only *TD_LSE* and *DFD_LSE* but also *TD_MLE* gave inconsistent lifetime estimates at the two excitation powers (e.g., *R*^*2*^ in red channel were 0.35, −1.77, and −2.32 for *TD_MLE, TD_LSE*, and *DFD_LSE*, respectively). In contrast, *flimGANE* provided much more consistent lifetime estimates regardless the excitation power (*R*^*2*^ was 0.75 in red channel; Fig. 3g).

### Quantifying Förster resonance energy transfer (FRET) efficiency in live MDA-MB-231 cells

Combined with the glucose FRET sensor, FLIM has been employed to image the glucose concentration in live cells^10,49^. However, depending on the lifetime analysis methods, the trend of FRET change can be skewed, especially when the donor lifetime change is very small (e.g., only 0.1–0.2 ns). Our glucose FRET sensor, termed CFP-*g*-YFP^50,51^, consisted of a glucose binding domain flanked by a cyan fluorescent protein (CFP) donor and a yellow fluorescent protein (YFP) acceptor (Fig. 4a). The overlap between CFP emission and YFP absorption leads to efficient dipole-dipole interactions. The CFP-*g*-YFP sensor-expressed MDA-MB-231 cancer cells were starved for 24 h before adding different amount of glucose to the cell culture (final concentrations: 0, 0.5, 1.0, 2.0, 5.0, 10.0, 15.0 mM). The *DFD* data were collected by the confocal scanning system from a 256 × 256-pixel area before and after the addition of glucose and then analyzed by *TD_LSE, TD_MLE, DFD_LSE*, and *flimGANE* methods to generate FLIM images based on the CFP donor decays (Fig. 4b). By proper selection of regions of interest (ROI) in imaging analysis, single cells were separated from each other and from the background noise (Supplementary Fig. 13; Supplementary Table 12). Thousands of lifetime data points (apparent lifetimes, *τ*_*a*_) were plotted in a histogram and the mean was extracted by Gaussian fitting, giving one representative donor lifetime for each glucose concentration (Supplementary Fig. 14). The energy transfer efficiency (*E*) was calculated based on the equation: *E* = *1* − *(τ*_*DA*_/*τ*_*D*_*)*, where *τ*_*D*_ and *τ*_*DA*_ were the representative CFP lifetimes before and after addition of glucose, respectively (Fig. 4c). Whereas only subtle lifetime changes were seen in the mean CFP donor lifetime (0.04–0.20 ns in Supplementary Table 12, which led to low FRET efficiencies around 0.02–0.07), *flimGANE*-derived FRET efficiencies were not only highly reproducible but also showing a general increasing trend at higher glucose concentrations. On the other hand, the lifetime of the acceptor (YFP) did not change upon the addition of glucose (Supplementary Fig. 15). Among the four methods, *DFD_LSE* failed to provide a FRET efficiency response curve due to its poor lifetime estimation in this experiment, thus being excluded from Fig. 4c.

When the intensity-based method, *E* = *1* *−* *(F*_*DA*_*/F*_*D*_*)*, was used to estimate *E*, the resulting response curve clearly deviated from the reasonable trend, possibly due to the artifacts such as photobleaching. At 2 mM glucose concentration, we could clearly see that the *flimGANE* image of CFP is the most similar to the *TD_MLE* image, but quite different from the *TD_LSE* and *DFD_LSE* images, in which there were many failed pixels (Fig. 4b; Supplementary Table 13). Although the *TD_MLE* images were similar to the *flimGANE* images, *TD_MLE*-derived FRET efficiencies had higher variations and showed an unrealistic, decreasing trend at higher glucose concentrations. In this demonstration, *flimGANE* not only gave a correct sensor response curve but also provided an analysis speed up to 2,800-fold faster than *TD_MLE* in reconstructing a FLIM image.

### Quantifying metabolic states in live HeLa cells

Autofluorescence of endogenous fluorophores, such as nicotinamide adenine dinucleotide (NADH), nicotinamide adenine dinucleotide phosphate (NADPH), and flavin adenine dinucleotide (FAD), are often used to characterize the metabolic states of cancer cells through metrics such as optical redox ratio (ORR)^52^, optical metabolic imaging index (OMI index)^53^ and fluorescence lifetime redox ratio (FLIRR)^54^. Since the fluorescence signatures of NADH and NADPH overlap, they are often referred to as NAD(P)H fluorescence in literature. NAD(P)H (an electron donor) and FAD (an electron acceptor) are metabolic coenzymes in live cells, whose autofluorescence intensity ratio reflects the redox states of the cells and the shifts in the metabolic pathways. However, intensity-based metrics (e.g., ORR) often suffer from wavelength- and depth-dependent light scattering and absorption issues when they are used to characterize the metabolic states of tumor tissues. In contrast, lifetime-based metrics (e.g., FLIRR) bypass these issues, revealing bias-free protein-binding activities of NAD(P)H and FAD^54^. As ORR and fluorescence lifetimes of NAD(P)H and FAD provide complementary information, they have been combined into the OMI index that can distinguish drug-resistant cells from drug-responsive cells in tumor organoids^55^.

Here we demonstrate that *flimGANE* provides rapid and accurate autofluorescence FLIM images of live HeLa cells. *DFD* data at two emission channels (NAD(P)H: 425–465 nm and FAD:511–551 nm) were collected by the ISS confocal scanning system (with 405 nm excitation) and the acquired data were analyzed by the four methods, generating both intensity and FLIM images (Fig. 5a, b). We adopted FLIRR (α_2_NAD(p)H_/α_1_FAD_) as a metric to assess the metabolic response of cancer cells to an intervention. It was found that *flimGANE*-derived FLIRR was highly correlated with its counterpart derived by *TD_MLE* (Fig. 5c, d; Supplementary Table 14). Since the NAD(P)H signals came from both the mitochondrial oxidative phosphorylation and cytosolic glycolysis and the FAD signals mainly originated from the mitochondria, image segmentation was often needed to deduce the relative contributions of oxidative phosphorylation and glycolysis to the cellular redox states and help quantify the heterogeneity of cell responses^54^. In our analysis, an intensity threshold was selected to isolate the mitochondrial regions from the rest of the cell area, where the nuclei were manually zeroed (Fig. 5e). Again, *flimGANE* outperformed the other three methods, generating results most similar to those reported in literature^54,56–58^, with FLIRR values of cancer cells around 0.2–0.4 (Fig. 5f). *TD_LSE* and *DFD_LSE* provided an incorrect representation, where the former was largely skewed by the low FLIRR values and the latter showed two unrealistic peaks. *TD_MLE* gave a distribution similar to that of *flimGANE*, but with a larger FLIRR peak value, due to the inaccurate estimate of NAD(P)H lifetime under the photon-starved conditions.

## Discussion

*flimGANE* addresses an unmet need for FLIM analysis—a computationally efficient, high-throughput and high-quality method for fluorescence lifetime estimation that works reliably even in the ultra-low-photon-count conditions (e.g., 50–100 photon counts per pixel; Fig. 1d). Among the cases studied in this report, *flimGANE* generated FLIM images with quality similar to those produced by the gold standard *TD_MLE*, but *flimGANE* clearly outperforms *TD_MLE* in barcode identification (Fig. 2), FRET characterization (Fig. 4), and metabolic state analysis (Fig. 5). We emphasize that here we intentionally acquired fluorescence data under the low- to medium-light conditions in order to compare the performance of the four methods. We found even the gold standard *TD_MLE* may not necessarily give consistent lifetime estimates under different excitation powers (Fig. 3g). It is thus critically important for users to understand the limitations of their lifetime analysis methods, especially when handling the low-count decays. Here we provide users with an alternative FLIM analysis approach, where the low-laser power requirement can reduce photobleaching and phototoxicity issues in delicate samples.

As FLIM finds more clinical applications such as in retinal imaging^59^ and tumor margin identification^60^ in recent years, it is necessary that we have an accurate and fit-free method to perform the lifetime imaging analysis. Through the use of convolutional and residual blocks, our model generates high-quality decays from low-photon-count inputs without introducing bias. The inference of lifetime is non-iterative and does not require parameter search to optimize the network performance. In this work, we evaluated the network performance using *in-silico* data, demonstrating that *flimGANE* can generate reasonable lifetime estimates with photon counts as low as 50 per pixel.

To the best of our knowledge, this is the first demonstration of a GAN model applied to reconstruct FLIM images. Since the use of Jensen-Shannon divergence as the objective function can cause problems such as vanishing gradients and mode collapse during GAN training, we incorporated Wasserstein metric in our model, which bypasses these issues by providing much smoother value spaces (Supplementary Figs. 16 and 17)^34^. We are continuing to explore the incorporation of other frameworks in our model, including the gradient penalty (WGAN-GP)^61^, the sequence generation framework (SeqGAN)^62^, and the context-aware learning^63^, that may in some instances provide more suitable approximate inference.

While *flimGANE* provides rapid, accurate, and fit-free FLIM analysis, its cost lies in the network training. In other words, *flimGANE* is particularly valuable for the FLIM applications where retraining is not frequently required. For instance, samples have similar fluorophore compositions (i.e., autofluorescence from metabolites in patient-derived organoids) and the IRF of the imaging system seldom changes. Different training datasets were employed to train the model separately that eventually led to the reliable results shown in Figs. 1–5 (Supplementary Table 1). A primary reason to retrain the model is due to the change of IRF (Supplementary Fig. 18). Whenever a different laser source is chosen for excitation, the filters are replaced, or the optics system is realigned, the IRF can also change, and the network should be retrained. For an entirely new imaging system, it can take up to 500 h to fully train the network with a lifetime range of 0.1–10.0 ns (two components, *τ*_*1*_ and *τ*_*2*_) and a pre-exponential factor range of 0.0–1.0 (*α*_*1*_). However, if we know the range of lifetime on our samples (e.g., 1.3–4.0 ns for the two lifetime components in barcode identification and 0.5–5.0 ns in live HeLa cell studies), a smaller training dataset can be employed to speed up the training process (e.g., 19 h in Supplementary Table 1).

Transfer learning^64^ from a previously trained network for handling a new sample can speed up the convergence of the learning process, and even potentially enhance overall performance. However, this is neither a replacement nor a required step for the entire training process. After running a sufficiently large number of training iterations for the generator (>2000), the optimal network can be selected when the validation loss no longer decreases. An essential step in generating a reliable FLIM image by our network is the accurate alignment of the fluorescence decay with respect to its corresponding IRF. A multistage preprocessing step, termed CoME (Supplementary Fig. 9), is employed to guarantee such an alignment. When running an IRF fluctuation test, a quality of estimate (*G*-quality score) based on known samples, and a discriminability test, we clearly show the advantages of *flimGANE* over other methods (Supplementary Figs. 19–21).

Taken together, our work represents an important step forward towards real-time and super-resolution^65,66^ FLIM. In fundamental biological research, further development of *flimGANE* will enable monitoring of fast binding kinetics and molecular transport dynamics inside live cells^67^. In medicine, *flimGANE* can provide rapid identification of tumor-free margin during tumor surgery^60^ and investigation of disease progression in the retina^59^. We envision that our method will soon replace *TD_MLE* and *TD_LSE* analysis packages in some commercial FLIM systems.

## Methods

### Structure of dataset

The dataset is composed of training data and testing data. Both training and testing data can be obtained either by a Monte Carlo (MC) simulation with the parameters or by acquiring experimental data from the ISS Alba v5 confocal microscopic system. The experimental data is the fluorescence decay histogram matrix of dimension *n* by *n* by *b* (*n* is the image size, which is either 256 or 512; *b* is the number of histogram bin size, which is 256 in this work).

### Structure of *flimGANE*

The *flimGANE* consists of a generator, a discriminator, and an estimator. The generator is composed of a convolutional block, a multi-task layer associated with the rectified linear unit (ReLU) activation function, a decoding layer associated with the tanh activation function, and a residual block implicitly. The discriminator is composed of four layers of neural networks. All the layers are fully connected layers composed of 128, 64, 8, and 1 node. The first three layers are associated with the sigmoid activation function, and the last one used a linear function. The estimator begins with two fully connected neural networks with 128 and 64 nodes, respectively, for incoming inputs, followed by the concatenation layer and a multitask layer associated with ReLU activation function (for further details, see Supplementary Methods).

We trained *flimGANE* with three-stage processes: generative model training, estimative model training, and *flimGANE* combination training. In generative model training, we adopt the Wasserstein GAN algorithm, where the generator and the discriminator are trained with Wasserstein loss. In estimative model training, the estimator is trained with the mean-squared error cost function. In *flimGANE* combination training, a well-trained generator and estimator are combined and trained with the mean-squared error cost function. The Adam optimizer is applied to the generator by setting the learning rate as 1 × 10^−4^. The RMSprop optimizer is applied to the discriminator by setting the learning rate as 5 × 10^−5^. The Adam optimizer is applied to the estimator by setting the learning rate as 0.001 (for further details, see Supplementary Methods).

### Evaluation metrics

To compare the proposed methods with other existing algorithms, we utilized several metrics, including execution time, mean-squared error for pixel-wise comparison, peak signal-to-noise ratio, structural similarity index, and visual information fidelity for the quality of the FLIM image with respect to the reference FLIM image (for further details, see Supplementary Methods).

### Statistics and reproducibility

Paired *t* tests and Kolmogorov-Smirnov tests were used for statistical comparisons of data. All data presented in the text are shown as mean ± standard deviation or mean ± standard deviation error, which are specified in each section. The significance level was set to 0.05 (* <0.05, **<0.001). Sample sizes and numbers are indicated in detail in each figure caption and main text. Exclusion criteria, if applied, are specified in each corresponding section.

### Reporting summary

Further information on research design is available in the Nature Research Reporting Summary linked to this article.

## Supplementary information

Supplementary Information

Description of Additional Supplementary Files

Supplementary Data 1

Reporting Summary

## Data Availability

Data behind Figs. 1e, f, 2c–e, 3c, and 4c are available in Supplementary Data 1. Data behind Figs. 3g and 5c, f are available at 10.6084/m9.figshare.17019065. Any other data generated in this study are available from the corresponding author on reasonable request.

## Code availability

All source codes, which can be obtained from https://github.com/NinaYIC/flimGANE, are available from the corresponding author on reasonable request.
