# Supplementary material for: Generative adversarial network enables rapid and robust fluorescence lifetime image analysis in live cells
Source: Commun Biol. 2022 Jan 11;5:18. doi: 10.1038/s42003-021-02938-w (PMC8752789; doi:10.1038/s42003-021-02938-w)
Supplement: Supplementary file 2 — Description of Additional Supplementary Files [file 42003_2021_2938_MOESM2_ESM.pdf]

Description of Additional Supplementary Files

File name: Supplementary Data 1

Description: The source data of Figure 1e, Figure 1f, Figure 2c-e, Figure 3c, and Figure 4c.
