# Supplementary material for: Generative adversarial network enables rapid and robust fluorescence lifetime image analysis in live cells
Source: Commun Biol. 2022 Jan 11;5:18. doi: 10.1038/s42003-021-02938-w (PMC8752789; doi:10.1038/s42003-021-02938-w)
Supplement: Supplementary file 4 — Reporting Summary [file 42003_2021_2938_MOESM4_ESM.pdf]

nature research

Corresponding author(s): Hsin-Chih Yeh

Last updated by author(s): Nov 15, 2021

Reporting Summary

Nature Research wishes to improve the reproducibility of the work that we publish. This form provides structure for consistency and transparency in reporting. For further information on Nature Research policies, see our Editorial Policies and the Editorial Policy Checklist.

Statistics

For all statistical analyses, confirm that the following items are present in the figure legend, table legend, main text, or Methods section.

n/a	Confirmed	
☐	☒	The exact sample size (*n*) for each experimental group/condition, given as a discrete number and unit of measurement
☐	☒	A statement on whether measurements were taken from distinct samples or whether the same sample was measured repeatedly
☐	☒	The statistical test(s) used AND whether they are one- or two-sided<br>*Only common tests should be described solely by name; describe more complex techniques in the Methods section.*
☒	☐	A description of all covariates tested
☐	☒	A description of any assumptions or corrections, such as tests of normality and adjustment for multiple comparisons
☐	☒	A full description of the statistical parameters including central tendency (e.g. means) or other basic estimates (e.g. regression coefficient) AND variation (e.g. standard deviation) or associated estimates of uncertainty (e.g. confidence intervals)
☐	☒	For null hypothesis testing, the test statistic (e.g. *F*, *t*, *r*) with confidence intervals, effect sizes, degrees of freedom and *P* value noted<br>*Give P values as exact values whenever suitable.*
☒	☐	For Bayesian analysis, information on the choice of priors and Markov chain Monte Carlo settings
☒	☐	For hierarchical and complex designs, identification of the appropriate level for tests and full reporting of outcomes
☒	☐	Estimates of effect sizes (e.g. Cohen's *d*, Pearson's *r*), indicating how they were calculated

Our web collection on statistics for biologists contains articles on many of the points above.

Software and code

Policy information about availability of computer code

Data collection	We used a Alba v5 (ISS Inc.), which was controlled by the VistaVision software, to acquire all data.
Data analysis	Deep learning models reported in this work used standard libraries that are available in TensorFlow v1.12.0 (Google Inc.). The custom codes were written in Python v3.6.5 (Anaconda modified version). All the software was developed and executed in the Microsoft Windows 10 operating system. For the fluorescent barcode bead selection, we register and assign an ID number for each one using ROI manager in ImageJ (http://imagej.nih.gov/).

For manuscripts utilizing custom algorithms or software that are central to the research but not yet described in published literature, software must be made available to editors and reviewers. We strongly encourage code deposition in a community repository (e.g. GitHub). See the Nature Research guidelines for submitting code & software for further information.

Data

Policy information about availability of data

All manuscripts must include a data availability statement. This statement should provide the following information, where applicable:

- Accession codes, unique identifiers, or web links for publicly available datasets
- A list of figures that have associated raw data
- A description of any restrictions on data availability

Data behind Figs. 1e, 1f, 2c-e, 3c, and 4c are available in Supplementary Data 1. Data behind Figs. 3g, 5c, and 5f are available at https://doi.org/10.6084/m9.figshare.17019065. Any other data generated in this study are available from the corresponding author on reasonable request.

Field-specific reporting

Please select the one below that is the best fit for your research. If you are not sure, read the appropriate sections before making your selection.

☒ Life sciences ☐ Behavioural & social sciences ☐ Ecological, evolutionary & environmental sciences

For a reference copy of the document with all sections, see nature.com/documents/nr-reporting-summary-flat.pdf

Life sciences study design

All studies must disclose on these points even when the disclosure is negative.

Sample size	We have trained with 990k samples and tested the approach on 2 different labeled samples with images with 256x256 pixels and 256 time bins. All barcode beads, Convallaria and HeLa cells, autofluorescence FLIM images were taken with 512x512 pixels and 256 time bins. All glucose FRET sensor FLIM images were taken with 256X256 pixels and 256 time bins.
Data exclusions	No data were excluded from the manuscript.
Replication	We have tested the deep learning model on multiple images with different experiments.
Randomization	Training, validation and testing images were randomly selected.
Blinding	All the performance metrics testing of the deep learning model results were blindly performed on the images that were not included in the training or validation phase.

Reporting for specific materials, systems and methods

We require information from authors about some types of materials, experimental systems and methods used in many studies. Here, indicate whether each material, system or method listed is relevant to your study. If you are not sure if a list item applies to your research, read the appropriate section before selecting a response.

Materials & experimental systems

n/a	Involved in the study	
☒	☐	Antibodies
☐	☒	Eukaryotic cell lines
☒	☐	Palaeontology and archaeology
☒	☐	Animals and other organisms
☒	☐	Human research participants
☒	☐	Clinical data
☒	☐	Dual use research of concern

Methods

n/a	Involved in the study	
☒	☐	ChIP-seq
☒	☐	Flow cytometry
☒	☐	MRI-based neuroimaging

Eukaryotic cell lines

Policy information about cell lines

Cell line source(s)	HeLa, and MDA-MB-231 were obtained from the American Type Culture Collection (ATCC).
Authentication	All cell lines were previously published and well characterized by many investigators.
Mycoplasma contamination	All cell lines used in this paper tested negative for mycoplasma.
Commonly misidentified lines (See ICLAC register)	No commonly misidentified cell lines were used.
